# Monthly dalbavancin dosing for suppressive therapy: a pharmacokinetic estimation analysis and case series

**DOI:** 10.1128/aac.01092-25

**Published:** 2025-12-19

**Authors:** Wesley D. Kufel, Kristen Tudahl, Paul Hutson, Rahul Mahapatra, Warren Rose, Cecilia Volk

**Affiliations:** 1 State University of New York Binghamton School of Pharmacy and Pharmaceutical Sciences 14787, Binghamton, New York, USA; 2 State University of New York Upstate Medical University 12302, Syracuse, New York, USA; 3 School of Pharmacy, University of Wisconsin–Madison 15533, Madison, Wisconsin, USA; Providence Portland Medical Center, Portland, Oregon, USA

**Keywords:** suppression, pharmacokinetic, dalbavancin

## Abstract

Retained hardware/prosthetic infections frequently require antimicrobial suppression therapy. Treatment options are often limited by resistance, allergies, and dosing frequencies. Dalbavancin (DAL) is a potentially attractive option for suppression of gram-positive infections given its potential for infrequent dosing. However, optimal DAL suppression dosing is unknown. An *in silico* pharmacokinetic/pharmacodynamic simulation was performed to assess the predicted dalbavancin concentration resulting from suppressive regimens. Serum levels were deemed adequate if the *f*AUC_24_/MIC was above the PK target of 27.1. Patients at a U.S. medical center receiving DAL as suppressive therapy were reviewed. PK simulation of DAL dosed 1,500 mg monthly resulted in free serum concentrations above the PK target. Because many clinicians opt to initiate these regimens with two doses given 1 week apart, the next modeled regimen included this load, before initiating 1,500 mg monthly. This initial load did not significantly alter total drug exposure. The final simulated regimen was 1,000 mg monthly. With this simulation, the lower 95% CI *f*AUC_24_/MIC fell just below the PK target for an isolate at the breakpoint. Nine patients who received dalbavancin 1,500 mg monthly as suppressive therapy were reviewed. All had retained hardware and received DAL for a median 591 days, with 7 patients still receiving treatment and no reported suppressive therapy failure. Monthly 1500 mg dalbavancin dosing for suppressive therapy is supported by this case series and PK simulation data. An initial weekly loading dose appears unnecessary. Reducing the monthly dose to 1000 mg may also be appropriate for certain patients, though clinical data is needed to support this practice.

## INTRODUCTION

Infections associated with retained hardware, including prosthetic joints, are difficult to treat and frequently require long-term suppressive antimicrobial therapy (SAT) (1). The use of oral agents is often preferred, but can be hindered by antimicrobial resistance, long-term tolerability, and patient adherence concerns (2, 3). In contrast, long-term intravenous suppression is complicated by stability and safety issues associated with central lines and frequent dosing of most intravenous (IV) agents. Ideally, surgical removal of prosthetic materials can shorten the required duration of therapy; however, surgical interventions are not always feasible (4). In such cases, SAT is commonly used to prevent recurrence of these complex infections.

Dalbavancin (DAL) is a long-acting glycopeptide currently approved by many regulatory agencies, including the Food and Drug Administration, to treat acute bacterial skin and skin structure infections (ABSSSI) caused by specific gram-positive organisms. There are two dosing regimens approved to treat ABSSSIs with DAL: (i) 1,500 mg given as a single dose and (ii) 1,000 mg followed by 500 mg 1 week later, both demonstrating non-inferiority to the standard of care in randomized control trials (5, 6). Increasingly, DAL is being used off-label for infection types other than ABSSSI, including use for chronic suppression in patients with retained, infected hardware. DAL is an attractive option for SAT due to its favorable adverse effect profile and less frequent dosing. This produces cascading benefits such as potentially decreased healthcare costs, less outpatient infusion appointments, and increased medication adherence (7, 8). The use of DAL as suppressive therapy (DST) is limited to case reports, with DAL commonly dosed every 2 weeks for these patients (9–19). A recent pharmacokinetic (PK) estimation analysis of DAL dosing for prolonged treatment durations revealed that the dosing interval may be increased further without sacrificing PK/pharmacodynamic (PD) target attainment (20). However, the goals for treatment versus suppression may require different approaches.

This report evaluates the potential effectiveness and target attainment of monthly DAL dosing for long-term DST using a case series of patients treated with this regimen and supportive PK estimation analysis.

## MATERIALS AND METHODS

### Pharmacokinetic modeling and analysis

*In silico* PK simulation was performed to assess the predicted DAL concentrations that would result from a variety of suppressive dosing regimens. Regimens were modeled from previously described doses and dosing intervals used in DST (9–13) as well as those from the patient case series. Using the previously established model published by Carrothers et al., a data set of 1,000 hypothetical patients was generated using patient and model parameters as described in our prior study (20, 21). Briefly, NONMEM (v7.5, ICON, Dublin, Ireland) was used to simulate free plasma concentrations for these simulated patients with age, weight, creatinine clearance, and albumin included as covariates in the model. Ninety-three percent protein binding was assumed.

For each modeled regimen, the unbound 24-h area under the curve over the minimum inhibitory concentration (24-h *f*AUC/MIC), which has an established target for stasis of 27.1 derived from the neutropenic murine thigh infection model, was estimated for each individual day of the simulated treatment period (22). A dosing regimen was determined to have adequate drug exposure for DST if the 24-h *f*AUC/MIC was above the target immediately prior to the next dose. The free serum concentration was determined assuming that 93% of the DAL was protein bound (23). Two MIC benchmarks were used: (i) the breakpoint of 0.25 µg/mL established by the Clinical and Laboratory Standards Institute (CLSI) for *S. aureus* and (ii) the 90th percentile MIC (MIC_90_) of 0.06 µg/mL for *S. aureus* reported from worldwide surveillance studies for DAL (24–26). Given a preferred target 24-h ratio *f*AUC/MIC = 27.1, the target 24-h *f*AUC for an organism at the breakpoint is 6.8 µg × h/mL and for the MIC_90_ is 1.6 µg × h/mL.

### Study design and practice setting

In order to validate the clinical efficacy of the 1,500 mg monthly suppressive regimen, we evaluated a cohort of patients who received this dosing strategy. A single-center, retrospective, observational case series included patients who received DST between 1 January 2015 and 1 January 2025, at ID Associates, an ID clinic affiliated with the State University of New York (SUNY) Upstate University Hospital in Syracuse, NY. Patients 18 years of age and older were included if they received DST for at least 180 days (4). Patients receiving DST were excluded if they were pregnant, incarcerated, or received DAL for active infection treatment without DST. Institutional Review Board exemption (#2026461-1) was obtained from SUNY Upstate Medical University.

DST selection and use were at the discretion of the treating ID clinician based on clinical and patient-specific factors. Insurance authorization and out-of-pocket expenses were evaluated prior to DST initiation. In this patient cohort, healthcare insurance providers provided full coverage of DST with no out-of-pocket charges related to drug cost and administration. All patients received DAL in the ambulatory clinic infusion suite via peripheral IV catheter with administration over 30 min. No DAL therapeutic drug monitoring (TDM) was performed. DAL susceptibility testing (via gradient strip diffusion) at the institutional microbiology laboratory was not available and/or not performed at the time that these patients’ cultures resulted.

### Study outcomes

The primary outcome was hospitalization due to a recurrent infection with the isolated index bacteria that initially facilitated DST use. Secondary outcomes included microbiological recurrence, repeat surgical intervention due to index infection, and DST discontinuation.

### Definitions

DST refers to the intended use of DAL to prevent bacterial spread from an uncontrolled source that could not be cured due to patient factors (e.g., surgery intolerance, uncurable source, poor antibiotic penetration). This treatment exceeds the standard duration for the infection type (e.g., >3–6 months) (4, 27). Following DST initiation, the primary outcome of hospitalization was defined as hospitalization to SUNY Upstate University Hospital during DST due to a recurrence of the index infection. Microbiologic recurrence was defined as a repeat culture growing the same pathogen that was originally isolated while receiving DST. Repeat surgical intervention due to the index infection was defined as a surgical procedure at the same anatomical site that required DST therapy. DAL discontinuation was defined as a provider-initiated discontinuation due to adverse drug reaction, orthopedic hardware removal, and/or clinical judgment for suppression therapy discontinuation, if applicable.

### Data collection and statistical analysis

All data collection was performed by a single investigator trained in data collection using the electronic medical record. Study data were collected and managed using a Research Electronic Data Capture platform (REDCap). Descriptive statistics were performed using Microsoft Excel. Continuous data were presented using median and interquartile range (IQR). Categorical data were presented using number and percentage (%).

## RESULTS

### PK analysis

**Fig 1:**
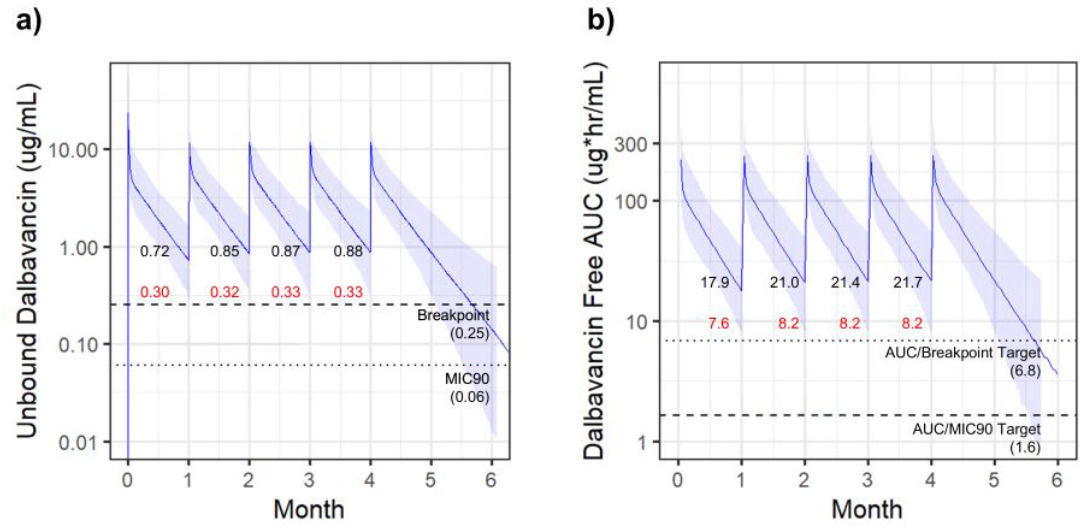
DAL 1,500 mg monthly. Simulated DAL exposure resulting from dosing of 1,500 mg monthly. Solid lines indicate the median value with shaded regions highlighting the 95% CI. Free serum concentrations indicated in (**a**) with 24-h *f*AUC in (**b**). PK targets are indicated with dotted lines. Trough values prior to each dose are indicated with median values (black) and lower 95% CI values (red).

The first modeled regimen was 1,500 mg administered once every 28 days (monthly), which represented the regimen for all patients in the case series described here. The PK simulation of this regimen resulted in free serum concentrations above both the breakpoint and MIC_90_ for the entire 95% CI, with the lowest resulting median trough level of 0.72 µg/mL (lower 95% CI = 0.3 µg/mL) (Fig. 1a). In addition, the resulting 24-h *f*AUC remained above both respective AUC/breakpoint and AUC/MIC_90_ targets for the entire 95% CI, with the lowest resulting trough level of 17.9 µg/mL (lower 95% CI = 7.6 µg/mL) (Fig. 1b). These results demonstrate 1,500 mg monthly dosing of DAL safely remains above multiple therapeutic targets, predicting adequate suppression of bacterial growth.

**Fig 2:**
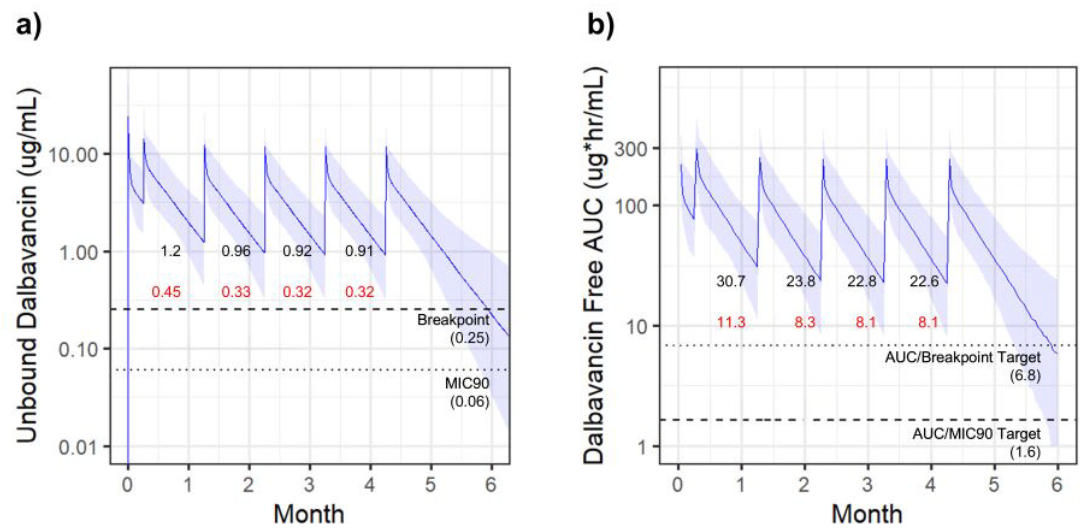
DAL1,500 mg monthly with an initial weekly loading dose. Simulated dalbavancin exposure resulting from dosing of 1,500 mg monthly with an initial weekly loading dose. Solid lines indicate the median value with shaded regions highlighting the 95% CI. Free serum concentrations indicated in (**a**) with 24-h *f*AUC in (**b**). PK targets are indicated with dotted lines. Trough values prior to each dose are indicated with median values (black) and lower 95% CI values (red).

Based on previously described DST use (12, 14, 15), many clinicians opt to utilize a loading dose strategy by initiating therapy with two 1,500 mg doses given 1 week apart, followed by more extended intervals. A second simulated regimen included this load before initiating DAL 1,500 mg monthly. This initial load did not significantly alter total drug exposure, with the lowest trough level at 0.91 µg/mL (lower 95% CI = 0.32 µg/mL) (Fig. 2a). The corresponding lowest trough of the 24-h *f*AUC was 22.6 µg/mL (lower 95% CI = 8.1 µg/mL) (Fig. 2b). While this loading dose led to predictably higher initial trough values, it did not improve the maintenance of levels above the AUC threshold and total drug exposure resulting from later doses and was similar to the regimen without the loading dose.

**Fig 3:**
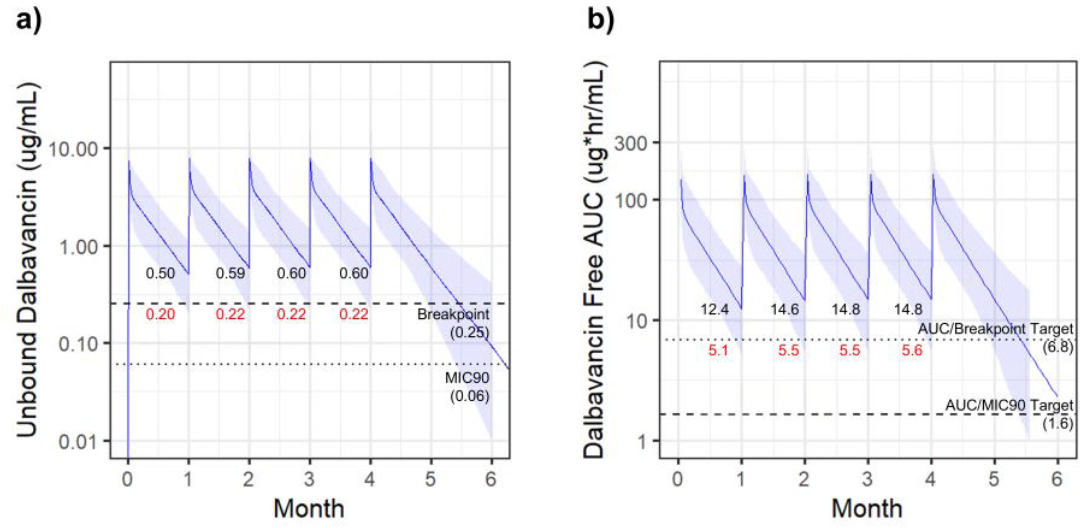
DAL 1,000 mg monthly. Simulated dalbavancin exposure resulting from dosing of 1,000 mg monthly. Solid lines indicate the median value with shaded regions highlighting the 95% CI. Free serum concentrations indicated in (**a**) with 24-h *f*AUC in (**b**). PK targets are indicated with dotted lines. Trough values prior to each dose are indicated with median values (black) and lower 95% CI values (red).

The final simulated regimen was 1,000 mg monthly. With this simulation, the lower 95% CI free serum levels fell below the MIC breakpoint, with the lowest trough of 0.5 µg/mL (lower 95% CI = 0.2 µg/mL) (Fig. 3a). The entire 95% CI maintained serum levels above the MIC_90_ target. Similarly, the lower 95% CI of the 24-h *f*AUC fell below the AUC/breakpoint target, while the entire interval remained above the AUC/MIC_90_ target (Fig. 3b).

### Case series

Between 1 January 2015 and 1 January 2025, nine patients received DST and were analyzed. Table 1 displays characteristics and outcomes among these patients. The median (IQR) of their demographics and laboratory findings included age of 67 (13) years, weight of 89.7 (36.6) kg, body mass index (BMI) of 27.4 (8.6) kg/m^2^, serum creatinine of 0.9 (0.15) mg/dL, creatinine clearance of 84 (73) mL/min/1.73 m^2^, and albumin of 3.7 (0.4) g/dL. With the exception of two patients over the age of 80 years, the patients were representative of the covariates included in the model, with patient weights, albumin levels, and creatinine clearance all falling within the modeled ranges. Five patients were male (55.6%). Two patients (22.2%) had chronic kidney disease stage 3 or higher, and four (44.4%) had immunocompromising conditions and/or received immunosuppressive medications. The most common indication for DST was prosthetic knee joint infections (55.6%) with *Staphylococcus epidermidis* (33.3%) and methicillin-resistant *Staphylococcus aureus* (MRSA) (33.3%) being the most common bacteria. Only two patients (22.2%) had polymicrobial infections and required an additional antimicrobial in addition to DST.

**TABLE 1:** DAL suppression therapy patient case summary^*b*^

Characteristic/ outcome	1	2	3	4	5	6	7	8	9
Age	33	55	94	74	68	61	61	67	84
Sex	Male	Male	Female	Male	Female	Male	Male	Female	Female
Weight (kg)	93.7	63.6	60.8	117.9	103.7	89.7	100.2	77.1	43.0
BMI (kg/m^2^)	30.3	19.6	24.5	21.7	36.9	30.1	30.8	27.4	21.3
Immunocompromising condition or medication	No	No	Yes	No	Yes	Yes	No	Yes	No
Chronic kidney disease stage 3 or higher	No	No	No	No	No	No	No	Yes	Yes
SCr (mg/dL)	0.75	0.86	0.91	0.99	0.77	0.84	0.9	2.0	1.3
CrCl (mL/min/1.73 m^2^)	120	120	34	80	84	90	110	37	27
Albumin (g/dL)	4.4	3.7	3.7	3.3	4	3.9	4	3.6	3.6
Treatment regimen prior to DST	Daptomycin, rifampin	Daptomycin	Dalbavancin	Dalbavancin	Cefazolin	Vancomycin	Dalbavancin	Linezolid	Vancomycin, rifampin
Indication for DST	Tibia and fibula hardware infection	Recurrent MRSA bacteremia with prosthetic aortic valve	Knee PJI	Lumbar spine hardware infection	Knee PJI	Knee PJI	Pelvic ring hardware infection	Knee PJI	Knee PJI
Bacteria targeted for DST	*Staphylococcus epidermidis*	MRSA	*Staphylococcus epidermidis*	*Corynebacterium striatum*	MSSA	*Staphylococcus epidermidis*	*Corynebacterium striatum*	MRSA	MRSA
Polymicrobial infection and other suppression antimicrobial	N/A	*Candida parapsilosis,* fluconazole	N/A	*Morganella morganii*, ciprofloxacin	N/A	N/A	N/A	N/A	N/A
DST Dosing Regimen	1,500 mg monthly	1,500 mg monthly	1,500 mg monthly	1,500 mg monthly	1,500 mg monthly	1,500 mg monthly	1,500 mg monthly	1,500 mg monthly	1,500 mg monthly
Duration of DST (days)^*a*^	637	617	994	591	327	360	396	1,273	470
Hospitalization due to recurrent or new index infection	No	No	No	No	No	No	No	No	No
Microbiological recurrence	No	No	No	No	No	No	No	No	No
Repeat surgical intervention required due to index infection	No	No	No	No	No	No	No	No	No
Rationale for DST discontinuation (if applicable)	Hardware removed	Still active	Still active	Still active	Still active	Still active	Still active	Hardware removed	Still active
DST-attributed adverse effects	None	None	None	None	None	None	None	None	None

^*a*^ Duration as of 31 May 2025 for patients where dalbavancin suppression therapy is still active.

^*b*^ BMI, body mass index; CrCl, creatinine clearance; DST, dalbavancin suppression therapy; kg, kilograms; MRSA, methicillin-resistant *Staphylococcus aureus*; MSSA, methicillin-susceptible *Staphylococcus aureus*; N/A, not applicable; SCr, serum creatinine.

All patients received DAL 1,500 mg IV monthly with a median (IQR) DST duration of 591 (241) days at the time of data collection. Seven patients were still receiving DST as of the time of this writing, with a range of 11 to 33 months of treatment. For the primary outcome, no patients experienced hospitalization due to a recurrent infection with the isolated index bacteria. Furthermore, no patients experienced microbiological recurrence or repeat surgical intervention due to the index infection. DST was discontinued in two patients after 21 and 42 months of therapy because surgical removal of orthopedic hardware no longer warranted DST. No patients experienced any DST-attributed adverse effects and/or intolerances resulting in dosing regimen modifications and/or discontinuation.

## DISCUSSION

This manuscript provides both clinical and PK evidence supporting the use of DAL 1,500 mg monthly for long-term infection suppression. Based on this simulation, this regimen maintains serum concentrations above the *f*AUC/MIC target for the entire dosing interval. To our knowledge, this is the first study evaluating the PK/PD target attainment of suppressive DAL regimens. Previous reports of DAL use for suppressive therapy are limited, with most studies utilizing biweekly dosing regimens (9–11, 13). Pallotto et al. previously reported a case series of six patients treated with a monthly DAL regimen; however, these patients all received an initial weekly loading dose, which appears to be unnecessary based on our simulations (12). To our knowledge, this is the first report of a monthly DAL regimen used for infection suppression. The patients included in this case series also had a variety of infection etiologies, indicating the potential for widespread use of DST for several gram-positive infections.

The long-acting lipoglycopeptides, dalbavancin and oritavancin, offer an attractive option for use as gram-positive chronic suppressive antimicrobial therapy, but this strategy is not yet supported by robust clinical use data. Of the two options, DAL has more extensive published use and data as a suppressive regimen. However, the infection sources, dosage and dosing intervals, and duration of therapy exhibit considerable variability in these reports. DST is most often reported for the suppression of deep-seated infections such as ventricular assist devices (9, 10, 13, 16, 17), prosthetic joints (11), prosthetic valves (15), endovascular grafts, septic arthritis, spinal implants, and chronic and vertebral osteomyelitis (18, 19, 28, 29). These infection types commonly involve biofilm, and DAL has notable anti-biofilm attributes *in vitro* and *in vivo* against MSSA and MRSA (30–33). The long terminal half-life of DAL (~14 days) enables its dosing as a suppression regimen. Most reports use DAL following a treatment phase with older antibiotics (e.g., vancomycin, daptomycin, cefazolin, etc.) with an initial IV dose of 1,500 mg; however, the remaining dosage(s) and dosing intervals for suppression are variable. These follow-up doses are 1,000 mg or 1,500 mg IV, with the latter being more commonly reported (16). Subcutaneous DAL administration for suppression has been reported (19), but this was not modeled in our study. The interval between doses is a considerable clinical question with reports ranging from weekly to every 4 weeks, with many studies using a biweekly dose. Our simulated models suggest that 1,500 mg biweekly dosing of DST provides supratherapeutic exposures well above the MIC breakpoint and MIC_90_. Therefore, dosing every 2 weeks is likely unnecessary for DST and likely to unnecessarily increase cost and patient inconvenience.

A significant barrier to guiding DST is the lack of consensus for an established PK/PD suppression target. This is a common flaw in the antimicrobial field as PK/PD for suppression regimens overall is not well established. We used the efficacy PK/PD target for stasis established from the mouse-thigh infection model of *f*AUC/MIC 27.1 (22), and extrapolated population MICs derived from either the breakpoint or MIC_90_ for *S. aureus*. We note that this estimate is highly conservative as SAT is designed to prevent bacterial recurrence following treatment for the acute phase of infection. This is consistent with the approach to treatment and DST use in the case series of this study. Multiple studies demonstrate clinical success when achieving stasis PK/PD targets for low-inoculum infections (34). For example, stasis PK/PD target attainment was found to be clinically effective in treating both ABSSSI and intra-abdominal infections (35, 36). While infections that require long-term suppression may not always be considered low-inoculum, suppressive therapy does not begin until after acute infection treatment and significant reduction in bacterial burden. This evidence supports the bacterial stasis PK/PD endpoint as a likely adequate target for suppression.

The use of DAL TDM also may be highly beneficial for suppression. When combined with the specific organism MIC, this can provide an improved precision approach to suppression to identify when the next dose is required. Using therapeutic drug monitoring to guide suppression dosing, Lafon-Desmurs and colleagues reported a median time of 57 days between doses (IQR 28–82 days) (29). The 1,500 mg monthly DAL dosing identified in our study is supported by the dosing interval range used in this TDM-guided study (29), but extended intervals beyond 1 month can be guided by TDM on an individual patient level (12, 29).

Overall, the simulated model results in this report demonstrate that 1,500 mg monthly dosing provides DAL exposure above MIC thresholds for the entire interval, allowing for improved stewardship of DST. Some studies have reported a loading dose strategy for DAL suppression of 1,500 mg on day 1 and another 1,500 mg 1 week later before initiating monthly dosing (12, 14, 15). Our model data suggest that this loading dose strategy is likely not necessary for suppression and would result in higher costs. The simulated dose of 1,000 mg monthly resulted in concentrations below the MIC thresholds; therefore, caution should be exercised when considering using this regimen for DST, especially when potential unforeseen circumstances could lead to delays in the typical monthly administration (e.g., infusion appointment rescheduling/cancelation for various reasons, insurance barriers, etc.). The 1,000 mg monthly regimen may be more appropriate for patients with renal impairment where slower drug clearance increases drug retention or for patients with body weight on the lower end of our model simulation, as lower volume of distribution and slower drug clearance may sustain levels above the PK/PD target over this duration. Finally, our study used population MICs for DAL as benchmarks, and most gram-positive organisms, especially *S. aureus*, fall well below the MIC_90_ and breakpoint used. Clinicians can extrapolate the 1,000 mg monthly PK simulations using the specific organism MIC to identify whether monthly dosing would be adequate to maintain DAL concentration above the MIC.

This study has several notable limitations. The first is the small size of the included case series of nine patients. Overall, there are limited reports of DAL use for suppression, and even fewer with monthly dosing. There is a significant need for larger observational studies or, ideally, randomized controlled trials in this space to establish clinical efficacy. Another notable limitation is the lack of clarity regarding PK/PD targets for infection suppression, as discussed above. As a conservative estimate, we opted to use established treatment PK/PD targets in this work. This target was based on MIC distributions and the breakpoint for *S. aureus*, so it may be difficult to extrapolate the findings to other gram-positive organisms. In addition, this study used a protein binding value of 93%, as described in the package insert; however, there are recent data suggesting that the true protein binding may actually be higher (37). Despite these limitations, it is noteworthy that patients experienced high success (clinical, microbiologic, and safety outcomes) with DST that was supported by our follow-up modeling simulations of the dose used.

In conclusion, this report provides evidence supporting the use of DAL 1,500 mg monthly dosing for long-term infection suppression. In comparison to the more frequently utilized biweekly dosing, this regimen has the potential to decrease costs for the patient while also potentially increasing adherence. This treatment option is especially appealing for patients who are poor candidates for oral therapy, based on resistance patterns or adherence concerns, or cannot manage complicated daily intravenous therapy. Furthermore, the use of TDM may be beneficial to further refine extended dosing for DST. Additional data are needed in this area to establish clinical efficacy and safety.
